# Histogram analysis of dynamic contrast-enhanced magnetic resonance imaging to predict extramural venous invasion in rectal cancer

**DOI:** 10.1186/s12880-023-01027-0

**Published:** 2023-06-08

**Authors:** Ke-xin Wang, Jing Yu, Qing Xu

**Affiliations:** Department of Radiology, First Affiliated Hospital of Nanjing Medical University, Gulou District, 300 Guangzhou Rd, Nanjing, 210029 Jiangsu China

**Keywords:** Rectal cancer, Extramural venous invasion, Dynamic contrast-enhanced magnetic resonance imaging, Histogram analysis, Prediction model

## Abstract

**Background:**

To explore the potential of histogram analysis (HA) of dynamic contrast-enhanced magnetic resonance imaging (DCE-MRI) in the identification of extramural venous invasion (EMVI) in rectal cancer patients.

**Methods:**

This retrospective study included preoperative images of 194 rectal cancer patients at our hospital between May 2019 and April 2022. The postoperative histopathological examination served as the reference standard. The mean values of DCE-MRI quantitative perfusion parameters (*K*^*trans*^, *K*_*ep*_ and *V*_*e*_) and other HA features calculated from these parameters were compared between the pathological EMVI-positive and EMVI-negative groups. Multivariate logistic regression analysis was performed to establish the prediction model for pathological EMVI-positive status. Diagnostic performance was assessed and compared using the receiver operating characteristic (ROC) curve. The clinical usefulness of the best prediction model was further measured with patients with indeterminate MRI-defined EMVI (mrEMVI) score 2(possibly negative) and score 3 (probably positive).

**Results:**

The mean values of *K*^*trans*^ and *V*_*e*_ in the EMVI-positive group were significantly higher than those in the EMVI-negative group (*P* = 0.013 and 0.025, respectively). Significant differences in *K*^*trans*^ skewness, *K*^*trans*^ entropy, *K*^*trans*^ kurtosis, and *V*_*e*_ maximum were observed between the two groups (*P* = 0.001,0.002, 0.000, and 0.033, respectively). The *K*^*trans*^ kurtosis and *K*^*trans*^ entropy were identified as independent predictors for pathological EMVI. The combined prediction model had the highest area under the curve (AUC) at 0.926 for predicting pathological EMVI status and further reached the AUC of 0.867 in subpopulations with indeterminate mrEMVI scores.

**Conclusions:**

Histogram Analysis of DCE-MRI *K*^*trans*^ maps may be useful in preoperative identification of EMVI in rectal cancer, particularly in patients with indeterminate mrEMVI scores.

**Supplementary Information:**

The online version contains supplementary material available at 10.1186/s12880-023-01027-0.

## Background

Extramural venous invasion (EMVI) is defined as the presence of tumor cells within blood vessels beyond the muscularis propria of the rectal wall in histology [1], which is an independent risk factor for local and distant recurrence [2, 3] and poorer overall survival of rectal cancer [4]. Traditionally, the gold standard to confirm EMVI status was postoperative pathology. Sometimes the obvious EMVI may be difficult to be identified via routine pathological analysis of the resection specimens because of the total destruction of extramural venous architecture by tumor tissue [5]. In addition, the obliteration and fibrosis of the invaded venous may appear after the neoadjuvant chemoradiotherapy (CRT) as mentioned before [6]. Therefore, these may result in false-negative in histopathology. The inaccurate EMVI assessment may influence the risk stratification and therapeutic decision-making [7]. Given its enormous significance, gaining a noninvasive and explicit diagnosis of EMVI status should be a priority for clinicians developing the individual therapeutic plan.

The magnetic resonance imaging-defined EMVI (mrEMVI) scoring system based on changes in vascular caliber and signal features was the primary imaging modality for specific and accurate assessment of EMVI status [8–10]. In addition, several studies suggested that mrEMVI has the potential to become an imaging biomarker for predicting histological grade, nodal stage, recurrence risk, and survival after neoadjuvant chemoradiotherapy (CRT) [11–15]. However, compared with postoperative pathology, the identification of EMVI based on conventional MRI can be inevitably misdiagnosed with a relatively low and wide range (28.2–62%) of sensitivity [14–16], especially among patients with indeterminate mrEMVI scores of score 2(possibly negative) and score 3 (probably positive) in small vessels (≤ 3 mm) perpendicular to the rectal wall [17]. Furthermore, the inflammation, edema, and fibrosis after neoadjuvant CRT may also increase difficulties in mrEMVI assessment [18, 19].

Recently, kinetic model quantitative parameters of dynamic contrast-enhanced MRI (DCE-MRI) has been frequently applied to describe characteristics of microcirculatory perfusion, and oxygenation in tumor via blood flow, capillary permeability, and permeability surface area in vivo [20–22]. Some studies have reported associations of DCE-MRI quantitative perfusion parameters with EMVI status in rectal adenocarcinoma [23–25] and reached the AUCs from 0.680 to o.856.

Histogram Analysis (HA), an emerging method that extracts non-visual imaging information and quantifies pixel intensity patterns within the tumor, indicates homogeneity, heterogeneity, asymmetry, vascularity, and necrosis [19, 23]. Features obtained from HA have been used to distinguish malignant tissues and microvascular invasion in the lung, glioma, colon, breast, and liver [26–29]. In rectal cancer, the applications of HA involve the prediction of locally advanced rectal carcinoma (stage T3-4 and/or N1-2), response after CRT, and overall survival [19, 30–32]. To our knowledge, the use of HA based on perfusion parameters of DCE-MRI to estimate EMVI status has not been well established in the literature.

Therefore, we aimed to achieve two goals. First, establish the prediction model of HA features from perfusion parameters based on DCE-MRI to identify EMVI status in rectal cancer patients. Second, further explore the diagnostic performance of this model in high-risk patients with suspicious positive mrEMVI findings (scores 2 and 3).

## Materials and methods

### Patients

This single-center study was approved by the Ethics of Committees of the First Affiliated Hospital of Nanjing Medical University of Jiangsu Province and informed consent for this retrospective study was waived.

This study enrolled 317 patients with consecutive rectal cancer between May 2019 and April 2022. Inclusion criteria were as follows: (1) Patients with pathologically confirmed rectal adenocarcinoma with any T and N stage, who underwent radical resection (R0) for rectal cancer within two weeks after high-resolution MRI and DCE-MRI examination, (2) Patients with no history of other pelvic cancers.

Totally 123 patients were excluded for the following reasons: (1) proven special histopathological type, including mucinous adenocarcinoma, signet ring cell carcinoma, and sarcomatous carcinoma, (2) received preoperative neoadjuvant CRT or anti-angiogenesis drugs treatment, (3) insufficient MRI quality for measurements with severe artifacts and mismatches between images, and (5) masses with extensive pelvic metastases. Ultimately, 194 patients were enrolled in the study.

### Reference standard

According to therapeutic principles, all patients underwent radical resection after MRI examination within two weeks in our tertiary care institution, using total mesorectal excision (TME) to remove the rectum and surrounding fatty tissue within mesorectal fascia or extended surgery (TME with adjacent visceral resection).

Histopathological information, including tumor differentiation grade, histological tumor stage, nodal stage, and EMVI result (present or absent), were obtained from pathology reports and confirmed by a pathologist with more than five years of experience in pathology. Patients were assigned to the EMVI-positive group or EMVI-negative group according to the pathological outcomes of surgical specimens.

### MRI acquisition

MRI scanning was acquired on a 3.0-T scanner (Magnetom Verio Tim; Siemens, Erlangen, Germany) with 16 elements of the pelvic phased-array coil. Non-enhanced MRI included: T1-weighted 2D turbo spin-echo imaging, sagittal, oblique axial and oblique coronal T2-weighted 2D turbo-spin-echo imaging, and long variable echo-trains diffusion-weighted imaging (b = 50 and 1000 s/mm^2^). Before injection of the contrast agent, noncontrast-enhanced T1-weighted 3D VIBE (Volume Interpolation Breath-hold Examination) gradient-echo images were performed. Then, the contrast agent (Omniscan, GE HealthCare, Milwaukee, WI;0.2 mL/kg) was bolus injected through the cubital vein with a flow rate of 2.5 mL/sec using an automated injector system (Stellant MR Injection System, Medrad, Germany). The detailed parameters for MRI sequences are summarized in Table 1.

**Table 1:** MRI sequences and parameters

	**T2-Weighted 2D**	**T2-Weighted 2D**	**T2-Weighted 2D**	**T1-Weighted 2D**	**DWI (b = 50, 1000 s/mm2)**	**T1-weighted 3D VIBE**
Orientation	Sagittal	Oblique axial	Oblique coronal	Oblique axial	Axial	Oblique axial
Sequence technique	TSE	TSE	TSE	TSE	Dual spin echo EPI	Spoiled gradient echo
Repetition time (msec)	4000	4550	4030	722	11,100	5.32
Echo time (msec)	99	99	129	11	91	1.81
Section thickness (mm)	3	3	3	3	5	3
Field of view (mm × mm)	250 × 250	220 × 220	250 × 250	220 × 220	360 × 300	280 × 250
Matrix	384 × 326	384 × 296	384 × 307	320 × 224	196 × 131	256 × 261
Acquisition time (min:s)	2:32	3:20	4:10	2:43	3:38	5:5

### MRI-defined EMVI detection

Monthly, preoperative images of all the patients were retrospectively revised by two radiologists (Reader 1 with four years of experience in rectal cancer imaging, and Reader 2 with 14 years of experience) to determine mrEMVI scores. Both radiologists were blinded to clinical information and postoperative histopathological findings. The consensus of the mrEMVI score from two radiologists was directly adopted. Discrepant scores were then delivered to a third radiologist with 27 years of experience in rectal MRI diagnosis for final decision. The mrEMVI scores were assessed using a 5-point scale ranging from 0 to 4 on MRI, suggested by Smith et al. [9]. The presence and degree of mrEMVI were categorized as score 0 (definitely negative), score 1(probably negative), score 2 (possibly negative), score 3 (probably positive) or score 4 (definitely positive), as shown in Supplementary Figure S1.

### DCE-MRI Post-processing

**Fig. 1:**
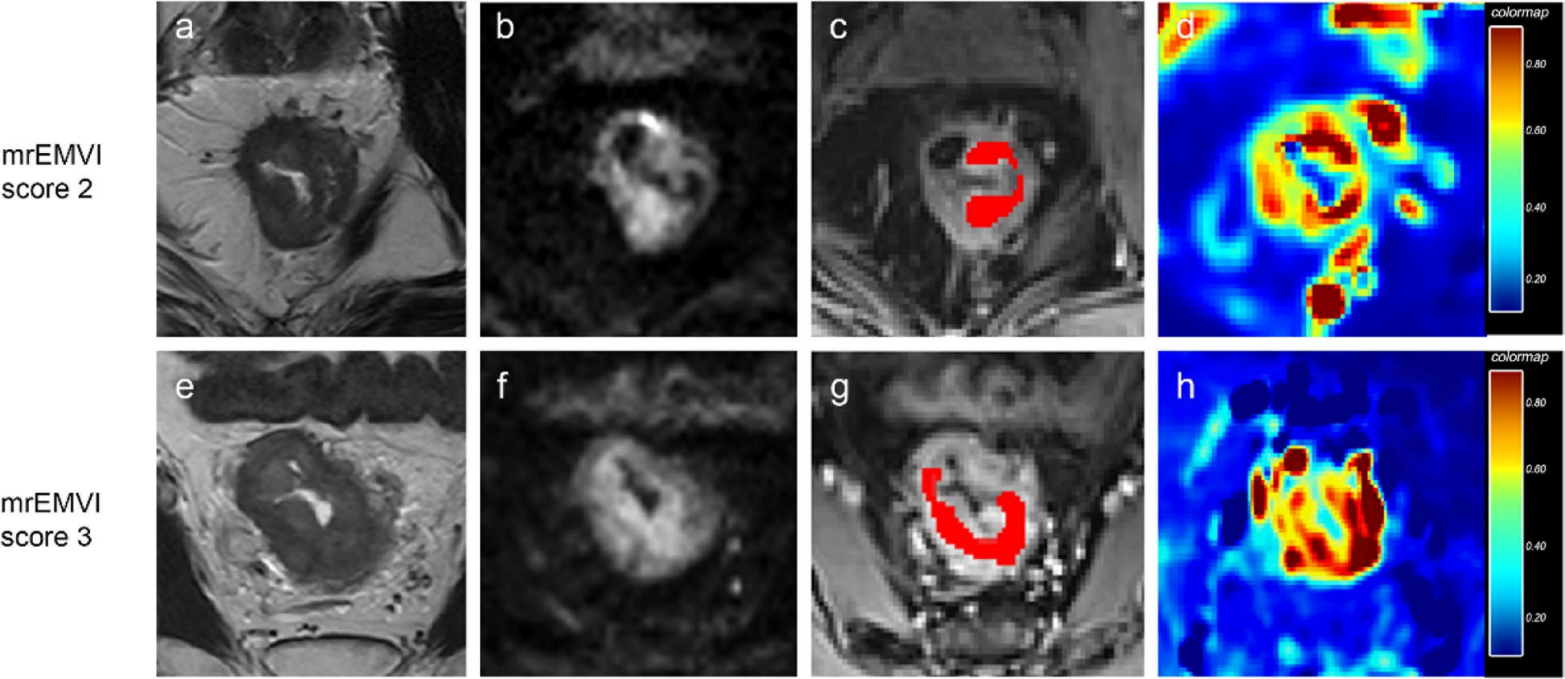
Image analysis of two male patients with mrEMVI score 2 and mrEMVI score 3 respectively. (a, e) Oblique axial T2-weighted (T2W) images. (b, f) Diffusion-weighted (DW) images at b = 1000 s/mm^2^. (c, g) Regions of interest (ROI) result on DCE-MRI. (d, h) Corresponding *K*^*trans*^ maps

The segmentation process was performed by Reader 1 and Reader 2 independently to ensure the reliability of measurements. Pharmacokinetic analysis was carried out using OmniKinetics (OK, GE Healthcare, China) based on the two-compartment extended tofts model in perfusion assessment. Personalized arterial input function (AIF) was obtained from the femoral arteries. Subsequently, time concentration series were calculated by contrast-enhanced time series. To avoid peripheral fat, artifacts, and blood vessels, regions of interest (ROIs) were manually drawn on DCE-MRI along the boundary of the tumor slice by slice to cover the whole tumor under the guidance of corresponding T2-weighted images and diffusion-weighted images (Fig. 1). All ROIs were merged for the volume of interest (VOI) of the whole tumor. Lastly, through the MatLab program (v. 2015b; MathWorks, Natick, MA), these mean values of kinetic model quantitative parameters (*K*^*trans*^, *K*_*ep*_ and *V*_*e*_) were calculated from all VOIs and other HA features (median, maximum, minimum, P10th, P90th, skewness, kurtosis, uniformity, energy, variance, and entropy) were extracted on the basis of these quantitative parameters. The final value considered in the statistical analysis was an average calculated from the values extracted by the two radiologists for each perfusion parameter and HA feature.

### Statistical analysis

All of the continuous variables were expressed as the mean ± standard deviations (SDs). Categorical variables were compared between the EMVI-positive and EMVI-negative groups using the χ2-test. The independent samples t-test was performed to compare the quantitative parameters and HA characteristics between the two groups. Multivariate logistic regression analysis using forward stepwise selection was applied to identify independent factors for pathologic EMVI status. Receiver-operating characteristic (ROC) curves were performed to assess the diagnostic efficacy of these independent factors and the combined model. The area under the curve (AUC) was calculated for each ROC. The DeLong test was conducted to compare AUCs between models. Interobserver agreement for each parameter of the two radiologists was determined by calculating intraclass correlation coefficients (ICCs) with 2-way random method (< 0.20, poor; 0.20–0.40, fair; 0.41–0.60, moderate; 0.61–0.80, good; and ≥ 0.81, excellent). All statistical analyses were performed using SPSS 23.0 (IBM Corp, NY). A two-sided *P* value < 0.05 was considered significant.

## Results

### Clinical-pathologic characteristics

The clinical-pathologic characteristics of 194 patients were summarized in Table 2. There were 136 (70.1%) patients in the pathologic EMVI-positive group and 58 (29.9%) patients in the EMVI-negative group. In total, 75 patients were marked with a mrEMVI score of 1, 59 patients with a mrEMVI score of 2, 42 patients with a mrEMVI score of 3, and 18 patients with a mrEMVI score of 4. Significant differences were observed in histological tumor stage and regional nodal metastases between the two groups (*P* = 0.001 and 0.028, respectively). Increased mrEMVI scores were significantly more frequent in the EMVI-positive group (*P* < 0.001). There were no significant differences in age, sex, histological grade, and mrCRM between the two groups.

**Table 2:** Clinical-pathologic characteristics of patients in the EMVI-positive and EMVI-negative groups

	**EMVI ( +) (*n* = 58)**	**EMVI (-) (*n* = 136)**	***P* value**
Age, mean ± SD	65.4 ± 9.8	68.2 ± 11.3	0.201
Sex			0.316
Male	31(53.4%)	62(45.6%)	
Female	27(46.6%)	74(54.4%)	
Histological grade			0.429
Well differentiated	11(19.0%)	17(12.5%)	
Moderately differentiated	29(50.0%)	79(58.1%)	
Poorly differentiated	18(31.0%)	40(29.4%)	
Histological stage			0.001*
T1-2	18(31.0%)	78(57.4%)	
T3-4	40(69.0%)	58(42.6%)	
Pathologic lymph node			0.028*
N0	25(43.1%)	82(60.3%)	
N1-2	33(56.9%)	54(39.7%)	
Tumor length, cm	5.05 ± 2.43	4.97 ± 3.22	0.474
mrEMVI Scores			0.000 *
1	12 (20.7%)	63 (46.3%)	
2	11 (19.0%)	48 (35.3%)	
3	17 (29.3%)	25 (18.4%)	
4	18 (31.0%)	0 (0%)	
mrCRM			
Negative (> 1 mm)	41 (70.7%)	112(83.4%)	0.069
Positive (≤ 1 mm)	17(29.3%)	24(17.6%)	

*P* values were derived from univariate association analyses (independent-sample t-test: age, tumor length; chi-squared test: sex, histological grade, pathologic lymph node, mrEMVI scores, and mrCRM

*EMVI* Extramural venous invasion, *mrEMVI* Magnetic resonance imaging defined-extramural vascular invasion, *mrCRM* Magnetic resonance imaging-predicted circumferential resection margin, *SD* Standard deviation

^*^P < 0.05

### DCE-MRI quantitative perfusion parameters and HA features between two groups

As shown in Table 3, the mean value, skewness, kurtosis, and entropy of *K*^*trans*^ in the EMVI-positive group were significantly higher than those in the EMVI-negative group (*P* = 0.013, 0.001, 0.000, and 0.002, respectively). The mean value and maximum of *V*_*e*_ were both significantly higher in the EMVI-positive group (*P* = 0.025 and 0.033, respectively). The mean value and all HA features of *K *_*ep*_ resulted in no significant difference between the two groups.

**Table 3:** Comparison of significant DCE-MRI quantitative perfusion parameters and HA features between the EMVI-positive and EMVI-negative groups

DCE-MRI parameter	HA feature	EMVI ( +) (*n* = 58)	EMVI (-)(*n* = 136)	*P* value
*K *^*trans*^/min^−1^	Mean	0.675 ± 0.278	0.564 ± 0.247	0.013 *
	Median	0.723 ± 0.412	0.672 ± 0.359	0.130
	Maximum	1.564 ± 0.973	1.429 ± 0.827	0.206
	Minimum	0.126 ± 0.079	0.113 ± 0.062	0.239
	P10	0.171 ± 0.080	0.112 ± 0.056	0.135
	P90	1.059 ± 0.663	0.981 ± 0.540	0.257
	Skewness	1.320 ± 0.338	0.897 ± 0.282	0.001*
	Kurtosis	2.341 ± 0.642	1.281 ± 0.562	0.000*
	Uniformity	0.359 ± 0.163	0.311 ± 0.140	0.057
	Energy	0.426 ± 0.159	0.482 ± 0.112	0.079
	Entropy	1.575 ± 0.532	0.832 ± 0.546	0.002*
	Variance	0.923 ± 0.412	0.872 ± 0.359	0.330
*V *_*e*_	Mean	0.446 ± 0.102	0.408 ± 0.117	0.025*
	Median	0.507 ± 0.253	0.473 ± 0.220	0.217
	Maximum	0.987 ± 0.574	0.795 ± 0.501	0.033*
	Minimum	0.121 ± 0.072	0.103 ± 0.062	0.231
	P10	0.187 ± 0.107	0.124 ± 0.084	0.108
	P90	0.886 ± 0.514	0.735 ± 0.498	0.436
	Skewness	2.153 ± 1.224	1.962 ± 0.986	0.319
	Kurtosis	1.751 ± 0.780	1.812 ± 0.936	0.236
	Uniformity	0.634 ± 0.278	0.583 ± 0.184	0.243
	Energy	0.784 ± 0.276	0.823 ± 0.314	0.210
	Entropy	1.862 ± 0.907	2.093 ± 1.045	0.126
	Variance	1.129 ± 0.547	1.037 ± 0.498	0.094
*K *_*ep*_ /min^−1^	Mean	1.487 ± 0.512	1.546 ± 0.423	0.321
	Median	1.193 ± 0.673	1.233 ± 0.872	0.107
	Maximum	2.512 ± 1.453	2.112 ± 1.122	0.116
	Minimum	0.395 ± 0.182	0.342 ± 0.156	0.532
	P10	0.415 ± 0.223	0.403 ± 0.198	0.232
	P90	2.173 ± 1.358	1.872 ± 0.104	0.354
	Skewness	3.369 ± 1.903	3.103 ± 1.554	0.426
	Kurtosis	2.112 ± 1.227	2.342 ± 1.754	0.576
	Uniformity	1.683 ± 0.891	1.532 ± 1.003	0.422
	Energy	0.690 ± 0.314	0.797 ± 0.336	0.066
	Entropy	1.094 ± 0.558	1.139 ± 0.683	0.073
	Variance	1.983 ± 1.127	1.763 ± 1.089	0.094

*EMVI* Extramural venous invasion, *K *^*trans*^ volume transfer constant between the blood plasma and the extracellular extravascular space, *V*_*e*_ Extracellular extravascular space volume fraction, *K *_*ep*_ Rate constant of contrast agent escape from the extracellular extravascular space into the plasma compartment

^*^P < 0.05

### Diagnostic performance of the combined prediction model

**Fig. 2:**
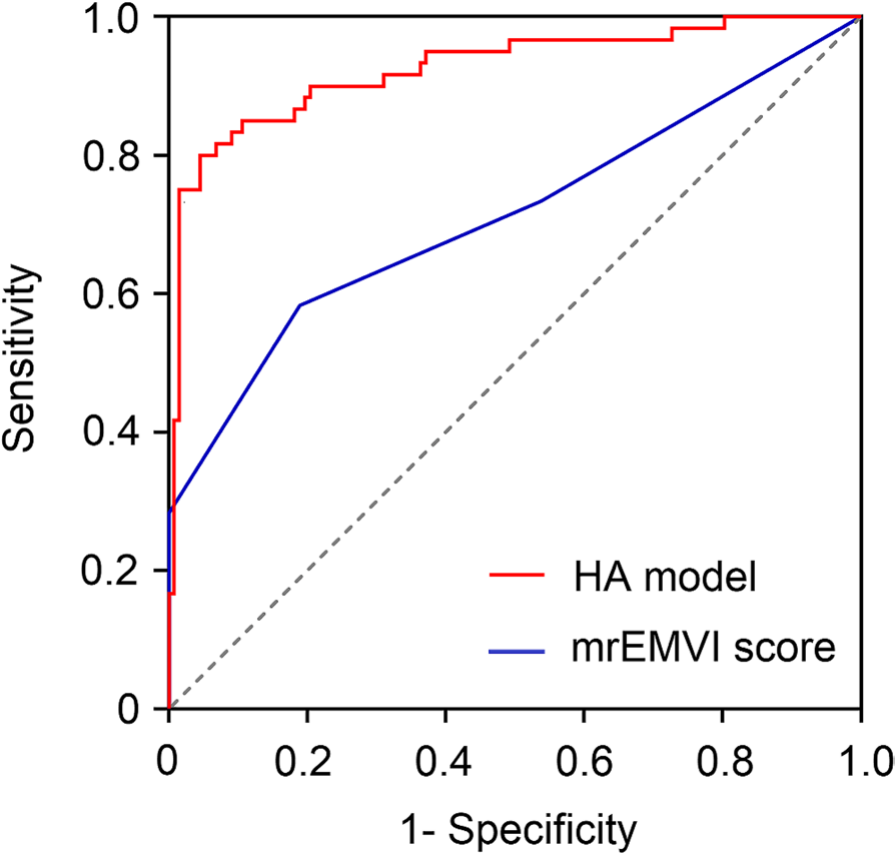
Comparison of ROC curves for HA model and the mrEMVI scoring system in identifying EMVI

**Fig. 3:**
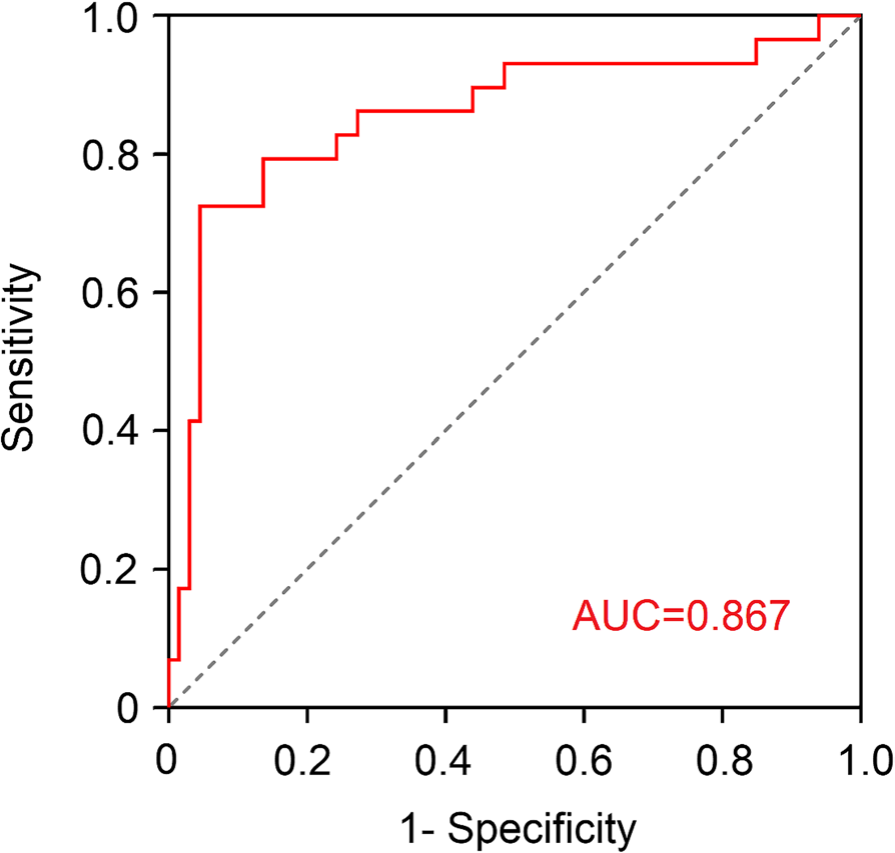
ROC curve for HA model in identifying EMVI in patients with indeterminate mrEMVI scores

Multivariate logistic regression analysis was conducted with significant clinical characteristics and HA features as covariables and pathologic EMVI status as the dependent variable. As displayed in Table 4, *K*^*trans*^ entropy (OR = 3.667, 95% CI 2.331–5.769, *P* < 0.001) and *K*^*trans*^ kurtosis (2.753, 95% CI 1.770–4.283, *P* < 0.001) were identified as independent predictors for the occurrence of EMVI. As shown in Fig. 2, the HA model combined *K*^*trans*^ skewness and kurtosis achieved a higher AUC of 0.926(95% CI, 0.881–0.791) with a sensitivity of 80.0% and specificity of 95.5%. The mrEMVI scoring system showed an AUC of 0.712, a sensitivity of 58.8%, specificity of 81.1%. Through the DeLong test, the AUC of the HA model was significantly improved compared with the mrEMVI scoring system (*P* < 0.001). As manifested in Fig. 3, the same HA prediction model further yielded the AUC of 0.867(95% CI,0.772–0.962), with a sensitivity of 72.4% and specificity of 93.2% in 101 patients with indeterminate MRI-defined EMVI scores of 2 and 3.

**Table 4:** Multivariate logistic regression analysis of combined prediction model

**Variables**	**Univariate analysis**			**Multivariate analysis**		
	**OR**	**95% CI**	***P* value**	**OR**	**95% CI**	***P* value**
*K*^*trans*^ mean	1.308	0.935–1.830	0.380			
*K*^*trans*^ skewness	1.339	0.552–3.243	0.027			
*K*^*trans*^ kurtosis	3.095	2.140–4.476	0.002	2.753	1.770–4.283	0.006
*K*^*trans*^ entropy	4.694	2.215–9.945	< 0.001	3.667	2.331–5.769	< 0.001
*V*_*e*_ mean	1.067	0.785–1.451	0.062			
*V*_*e*_ maximum	1.621	1.090–2.412	0.046			

*OR* Odds ratio, *CI* Confidence interval

### Interobserver variability evaluation

All kinetic perfusion parameters and HA features extracted from two sets of ROIs delineated separately by two radiologists showed good or excellent agreement (ICCs ranged from 0.773 to 0.906).

## Discussion

Preoperative identification of EMVI in rectal cancer assumes a key role in accurate risk stratification and treatment decision-making. Our findings demonstrated the potential of HA features from quantitative perfusion parameters based on DCE-MRI to preoperatively distinguish pathological EMVI status in rectal cancer patients. The *K*^*trans*^ kurtosis and *K*^*trans*^ entropy were identified as independent predictors for the occurrence of pathological EMVI. In addition, we demonstrated the good diagnostic performance of this combined HA model for diagnosing EMVI, particularly in subpopulations with indeterminate mrEMVI scores of 2 and 3.

The prior research elucidated that the presence of EMVI has been identified as an independent risk factor for recurrence risk and poor postoperative survival in rectal cancer [2, 4, 7]. However, consistent with previous findings [14–16], mrEMVI scores could not well satisfactorily coincide with postoperative pathological EMVI outcomes in this study. Subtle changes in signal features within small extramural vessels with slightly expanded contour and caliber cannot be easily distinguished, especially in rectal cancer lesions with mrEMVI score 2 (possibly negative) and score 3(probably positive) [9, 12].

We demonstrated that the lesions of the EMVI positive-group had significantly higher mean values of *K*^*trans*^ and *V*_*e*_ than the EMVI-negative group. Pathologically, the initial formation of EMVI is dependent on tumor angiogenesis, and then tumor cells invade into microvascular and eventually extend beyond the rectal wall [7, 13, 32]. These developing processes might be reflected by alterations of microcirculatory perfusion in tumor tissue. *K*^*trans*^ is the transfer rate from plasma to extracellular extravascular space (EES), which may correlate to tumor capillary permeability and angiogenesis. Higher *K*^*trans*^ represents greater microcirculation perfusion within tumor tissue [32]. *V*_*e*_ represents the fractional volume of the EES. The proliferation of tumor cells might reduce local permeability surface area, diminish microcirculatory perfusion and lead to microscopic necrosis, thus enlarging EES [23, 33]. Obliteration of the function of cell–cell adhesion molecules might also contribute to elevated *V*_*e*_ value [21, 34]. Yu et al. reported significantly higher *K*^*trans*^ and *V*_*e*_ in the EMVI-positive group than in the EMVI-negative group [25]. Chen et al. discovered that the mrEMVI-positive group had significantly higher *V*_*e*_ than the mrEMVI-negative group, but *K*^*trans*^ showed no significant difference [24]. Wei et al. demonstrated that *K*^*trans*^ was the independent predictor of EMVI in rectal cancer [35]. However, the mean value of *K *_*ep*_ did not show a significant difference between the EMVI-positive and EMVI-negative patients. The *K *_*ep*_ value is equivalent to *K*^*trans*^ / *V*_*e*_ and represents the rate constant of contrast agent escape from the EES into the plasma compartment. It was speculated that *K*^*trans*^ and *V*_*e*_ cannot increase unlimitedly with the increasing degree of vascularization and invasion depth of tumor cells [25]. In addition, with the increase of contrast agent concentration in tumor EES from plasma, the pressure difference between inside and outside microvessels decreases, which may diminish the diffusion rate of contrast agent from EES to plasma in a certain extent, that is, affect the *K *_*ep*_ [22, 24, 25].

Our investigation displayed significantly higher mean values of *K*^*trans*^ and *V*_*e*_ resulted suggestive of pathologic EMVI status. However, these two parameters were not identified as independent predictors of EMVI in rectal cancer through the multivariate logistic regression analysis. It was speculated that these two parameters might be mutually correlated and restricted [24]. Also, the mean values of *K*^*trans*^ and *V*_*e*_ might attenuate differences in perfusion performance between the regional highly vascularized areas of EMVI and other lesions.

Perfusion heterogeneity reflected by HA features obtained on DCE-MRI parameters may provide additional information to improve the diagnostic accuracy of EMVI in rectal cancer. In our study, patients in the EMVI-positive group tended to have statistically higher skewness, kurtosis and entropy of *K*^*trans*^ and maximum of *V*_*e*_ in the EMVI-positive group. Moreover, multivariate logistic regression analysis indicated that *K*^*trans*^ kurtosis and *K*^*trans*^ entropy acted as potential strong predictors of pathological EMVI status. The elevated heterogeneity, originates from more variable cellularity, disordered angiogenesis and variations in necrosis areas, which was proposed as critical characteristic of malignant lesions [36, 37]. Entropy reflects the intratumoral randomness of gray-level distribution. Higher entropy indicates greater heterogeneity, which was reportedly suggestive of higher histological tumor stage and poorer postoperative survival in various malignant tumors, including advanced breast cancer, hepatocellular carcinoma, and rectal cancer [28–30, 38]. Wilson et al. found the entropy as a potential strong predictor for microvascular invasion in hepatocellular carcinoma [29]. Kurtosis is a measure of the peakedness of the distribution of values in lesions. Wilson et al. also illustrated that greater imaging inhomogeneity might represent histopathological tumor heterogeneity and aggressiveness in hepatocellular carcinoma [29]. Liu et al. proposed that intratumoral heterogeneity might correlate with higher aggressiveness and pathological stages in rectal cancer [30]. Zhu et al. proposed that higher tumor angiogenesis permeability and blood perfusion could facilitate hematogenous metastasis and the formation of EMVI in gastric cancer [39]. Therefore, our results could be explained by the speculation that in the pathological EMVI-positive group, the primary lesions of rectal cancer may be more heterogeneous and aggressive, and local tumor area with hyper-perfusion and high angiogenesis permeability might be more conducive for intravascular tumor cells to protrude outside the intestinal wall, leading to the occurrence of EMVI. These findings realized the assessment of heterogeneity of microcirculation perfusion through signal gray-level distribution characteristics in rectal cancer lesions.

In addition, we evaluated the interobserver variability for HA features extraction. Lambin et al. introduced the radiomics quality score (RQS) in 2017 to ensure the standardization and homogenization of radiomics studies [40]. The aim of the RQS is to evaluate the methodological quality of radiomics-based investigations and identify high-quality results using the ICCs or Cohen’s kappa [41]. Our results indicated good or excellent agreements between the two radiologists for the delineation of whole-VOIs and the calculation of HA features based on multiple slices from DCE-MRI, to ensure the reliability and reproducibility of measurements.

Several limitations of this study should be noted. First, the retrospective nature of this study was a major limitation. Second, the selection of patients with mrEMVI scores of 2 and 3 for further assessment of the diagnostic efficacy of the combined model may have unavoidable selection bias. Third, these patients who received preoperative neoadjuvant CRT were excluded to avoid false-negative in this study. The reliable method of detecting EMVI in patients who undergo neoadjuvant therapy needs further research to confirm. Fourth, the relatively small sample size could compromise the generalizability and stability of our findings. A larger standard multicenter study is needed.

In conclusion, our study demonstrated that HA features obtained from DCE-MRI could help to identify the tumor biology of EMVI and achieve satisfactory radiologic-pathologic matching in rectal cancer patients, even in subpopulations with indeterminate mrEMVI scores of 2 and 3. These findings might be beneficial to preoperative risk stratification and therapeutic decision-making.

## Supplementary Information

**Additional file 1:** **Supplementary Figure S1.** Magnetic resonance imaging-defined EMVI scoring system. mrEMVI score 0: no vessels adjacent to areas of tumor penetration.mrEMVI score 1: minimal extramural stranding or nodular extension, but not in the vicinity of extramural vessels.mrEMVI score 2: stranding demonstrated in the vicinity of extramural vessels, but the vicinity of extramural vessels with normal caliber and no definite tumor signal within the vessel.mrEMVI score 3: intermediate tumor signal intensity apparent within vessels with contour and caliber slightly expanded.mrEMVI score 4: obvious irregular vessel contour or nodular expansion of vessel by definite tumor signal.

## Data Availability

The datasets used and/or analyzed during the current study are available from the corresponding author on reasonable request.

## Abbreviations

AUC: Area under the curve
DCE-MRI: Dynamic contrast-enhanced magnetic resonance imaging
EES: Extravascular-extracellular space
EMVI: Extramural vascular invasion
HA: Histogram analysis
ICC: Intraclass correlation coefficients
*K*_*ep*_: Rate constant of contrast agent escape from EES into the plasma compartment
*K*^*trans*^: Volume transfer constant between the blood plasma and EES
mrEMVI: MRI defined-extramural vascular invasion
ROC: Receiver operating characteristic
ROI: Region of interest
RQS: Radiomics quality score
*V*_*e*_: Extracellular extravascular space volume fraction
VOI: Volume of interest
