# Supplementary material for: Histogram analysis of dynamic contrast-enhanced magnetic resonance imaging to predict extramural venous invasion in rectal cancer
Source: BMC Med Imaging. 2023 Jun 8;23:77. doi: 10.1186/s12880-023-01027-0 (PMC10249234; doi:10.1186/s12880-023-01027-0)
Supplement: Supplementary file 1 — Additional file 1: Supplementary Figure S1. Magnetic resonance imaging-defined EMVI scoring system. mrEMVI score 0: no vessels adjacent to areas of tumor penetration.mrEMVI score 1: minimal extramural stranding or nodular extension, but not in the vicinity of extramural vessels.mrEMVI score 2: stranding demonstrated in the vicinity of extramural vessels, but the vicinity of extramural vessels with normal caliber and no definite tumor signal within the vessel.mrEMVI score 3: intermediate tumor signal intensity apparent within vessels with contour and caliber slightly expanded.mrEMVI score 4: obvious irregular vessel contour or nodular expansion of vessel by definite tumor signal. [file 12880_2023_1027_MOESM1_ESM.docx]

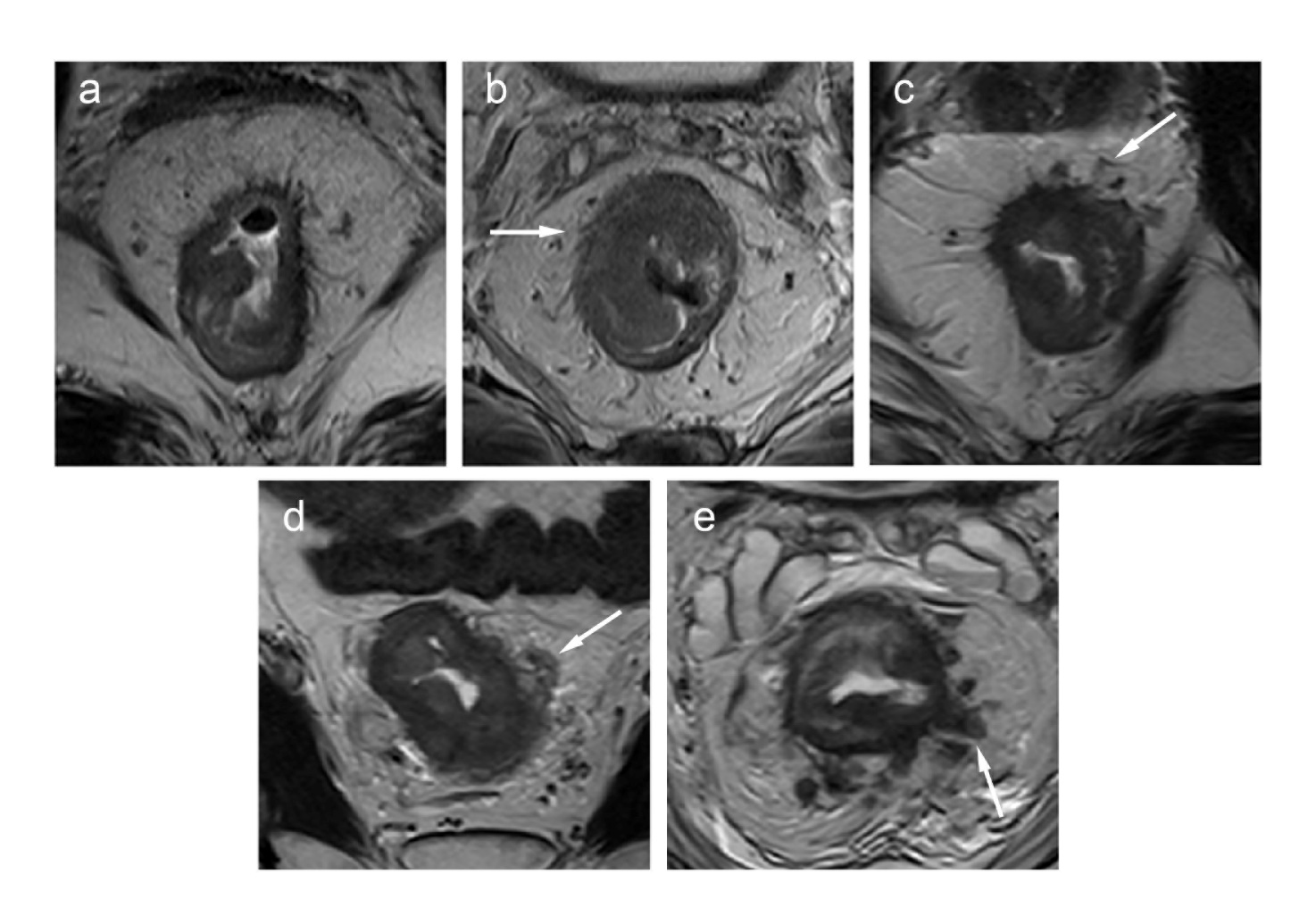


**Supplementary Figure S1.** Magnetic resonance imaging-defined EMVI (mrEMVI) scoring system (scores of 0,1 and 2 represent negative findings of EMVI and scores of 3 and 4 indicate positive findings of EMVI). (a) mrEMVI score 0: no vessels adjacent to areas of tumor penetration. (b) mrEMVI score 1: minimal extramural stranding or nodular extension, but not in the vicinity of extramural vessels. (c) mrEMVI score 2: stranding demonstrated in the vicinity of extramural vessels, but the vicinity of extramural vessels with normal caliber and no definite tumor signal within the vessel. (d) mrEMVI score 3: intermediate tumor signal intensity apparent within vessels with contour and caliber slightly expanded. (e) mrEMVI score 4: obvious irregular vessel contour or nodular expansion of vessel by definite tumor signal.
